# Supplementary material for: Maternal Intake of Either Fructose or the Artificial Sweetener Acesulfame-K Results in Differential and Sex-Specific Alterations in Markers of Skin Inflammation and Wound Healing Responsiveness in Mouse Offspring: A Pilot Study
Source: Nutrients. 2023 May 29;15(11):2534. doi: 10.3390/nu15112534 (PMC10255090; doi:10.3390/nu15112534)
Supplement: Supplementary file 1 [file nutrients-15-02534-s001.zip › nutrients-2347140-supplementary.pdf]

Supplementary Figure S1. Semi-quantitative scoring of inflammatory markers Il1β and CD68 (macrophages) and Tnfα immunostaining in the epidermis and dermis at D5 and D9 post-wounding in male and female offspring.

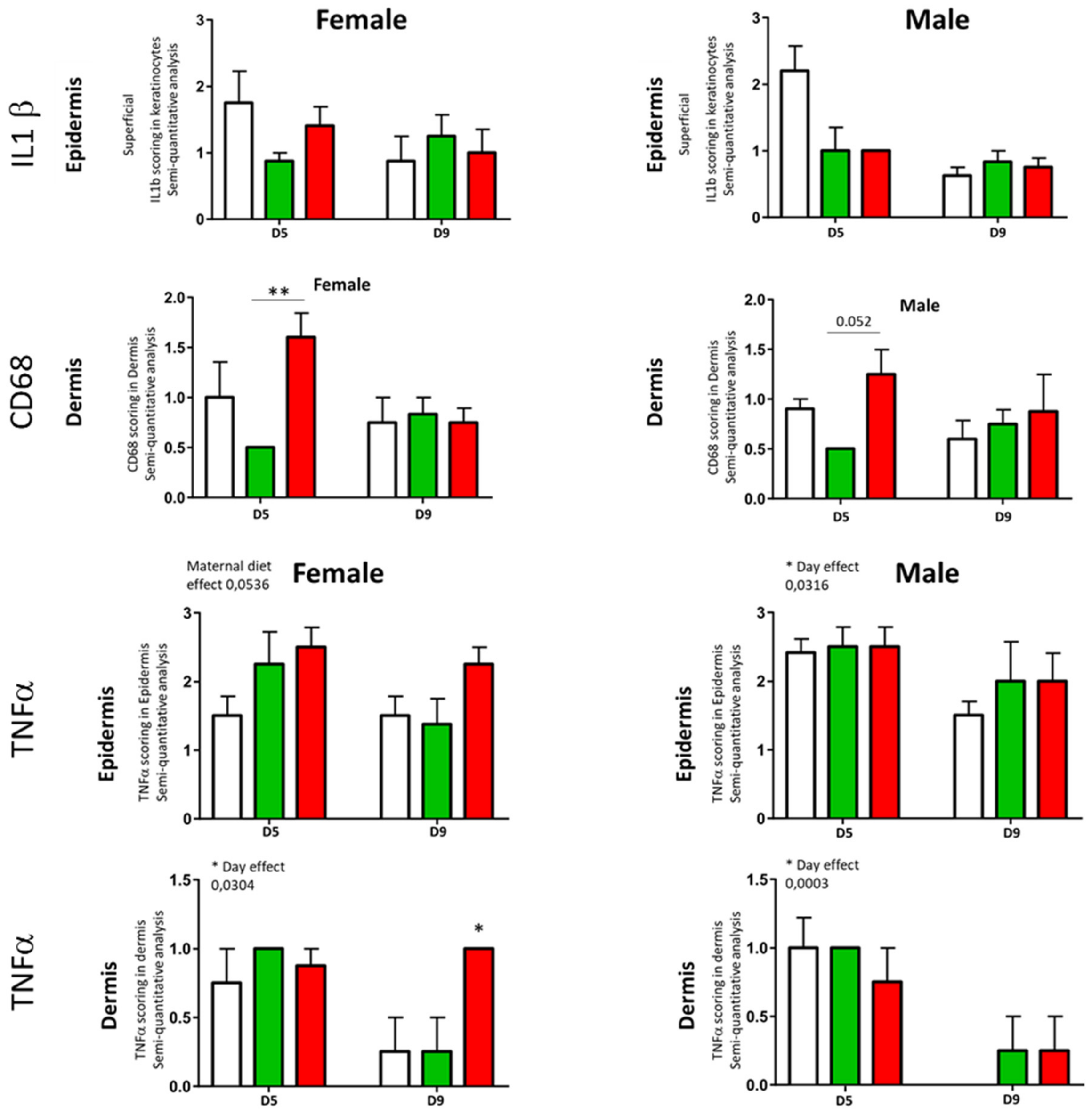

Supplementary Figure S2. CD-68 immunostaining in the epidermis and dermis at D5 and D9 post-wounding in male and female offspring.

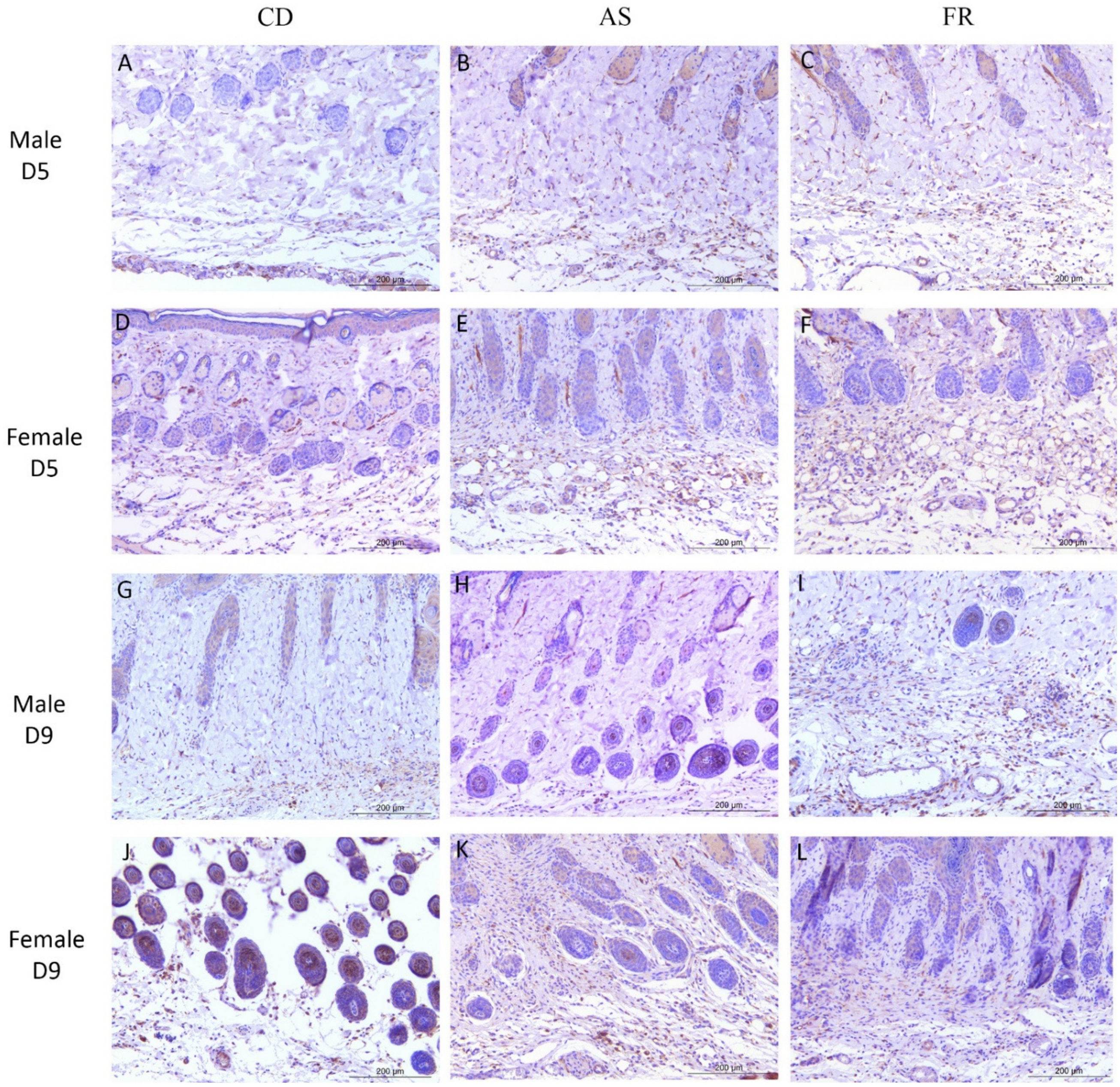

Table S1. Teklad Global 18% Protein Rodent Diet macronutrient composition.

Macronutrients	% kcal
Crude Protein	18.6
Fat (ether extract)	6.2
Carbohydrate (available)	44.2
Crude Fibre	3.5
Neutral Detergent Fibre	14.7
Ash	5.3
Calories from Protein	24
Calories from Fat	18

Calories from Carbohydrate	58
Energy Density	3.1 kcal/g

Table S2. Details of antibodies used in immunohistochemistry procedures.

Antibody	Reference	Dilution
Anti-IL1β	Abcam, ab9722	1:200
Anti-CD68	LS Bio, 32677	1:500
Anti-TNFα	Abcam, 1793	1:100
